# *Trypanosoma minasense* Parasitism in Free-Living and Captive Invasive Marmosets (Callitrichidae, Primates) in Seropédica, Rio de Janeiro, Brazil

**DOI:** 10.1007/s11686-026-01306-0

**Published:** 2026-06-01

**Authors:** Diogo P. Coimbra, Fernanda S. Nogueira, Diego M. Penedo, Cláudia B. Silva, Denise M. Nogueira

**Affiliations:** 1 Animal Biology PHD Program, Institute of Biological and Health Science, Universidade Federal Rural do Rio de Janeiro, Seropédica, Rio de Janeiro, Brazil; 2 Veterinary Medicine undergraduate, Institute of Veterinary Medicine, Universidade Federal Rural do Rio de Janeiro, Seropédica, Rio de Janeiro, Brazil; 3 Rio de Janeiro, Brazil; 4 Department of Parasitology, Institute of Veterinary Medicine, Universidade Federal Rural do Rio de Janeiro, Seropédica, Rio de Janeiro, Brazil; 5 Department of Genetics, Institute of Biological and Health Science, Universidade Federal Rural do Rio de Janeiro, Seropédica, Rio de Janeiro, Brazil

**Keywords:** *Callithrix*, Trypanosomatids, *Megatrypanum*, Morphometry, *18S rRNA*

## Abstract

**Purpose:**

Hemoparasitism by *T. minasense*, *T. devei*, and *T. cruzi* has been reported in *Callithrix jacchus* and *C. penicillata*, which are invasive marmoset species in Rio de Janeiro. We aimed to investigate parasitism by *Trypanosoma* sp. in marmosets inhabiting Seropédica municipality in Rio de Janeiro, as part of the health monitoring efforts due to the proximity of these primates to the local population and the Universidade Federal Rural do Rio de Janeiro/UFRRJ campus.

**Methods:**

Parasite species identification was performed through microscopy combined with PCR amplification, and DNA sequencing of ca. 700 bp fragment of the *18 S rRNA* gene. Blood samples were collected from twenty-five marmosets: twelve free-living individuals from UFRRJ campus and thirteen in captivity at CETAS-RJ.

**Results:**

Two individuals tested positive by microscopy for *Trypanosoma sp.*, with morphometry within the range for *T. minasense*. Ten individuals were positive by PCR, confirming it as a more efficient methodology for this hemoparasite detection. Sequences of the *18 S rRNA* showed similarity above 98% with *T. minasense*, clustering accordingly in the phylogenetic tree. Free-living marmosets presented a higher infection rate (58%) than captive animals (23%).

**Conclusion:**

The results suggest that captivity conditions may influence parasite transmission and fragmented landscapes maintain active trypanosomatid cycles, necessitating continuous surveillance in regions of increasing human-wildlife proximity.

**Supplementary Information:**

The online version contains supplementary material available at 10.1007/s11686-026-01306-0.

## Introduction

Marmosets are neotropical primates of the *Callithrix* genus (Mammalia: Primates), which comprises six species: *Callithrix kuhlii*, *C. flaviceps*, *C. geoffroyi*, *C. jacchus*, *C. aurita*, and *C. penicillata* [1], of which the last three occur in Rio de Janeiro state [2]. However, only *C. aurita* is native [3]. From the Northeast Brazil, *C. jacchus* is native of the states of Alagoas, Ceará, Paraíba, Pernambuco, Piauí, and Rio Grande do Norte [3]. *Callithrix penicillata* is native to central Brazil, and occurs in the states of Goiás, Tocantins, São Paulo, Minas Gerais, and Bahia [3]. Both are considered invasive species in Rio de Janeiro, introduced through animal trafficking [3, 4]. Invasive marmosets interbreed with *C. aurita*, yielding potentially fertile hybrids [3, 5]. *C. penicillata* has the highest potential impact due to shared environmental fitness patterns with the native species [6]. Additionally, phylogenetic proximity may facilitate parasites transmission [7].

While thirty-seven parasites species are known to infect callitrichids [8, 9] only two records exist for *C. aurita*: *Primasubulura jacchi*, a common primate nematode [10, 11], and *Prosthenorchis elegans* [9], a highly pathogenic and leading cause of death in captivity [12, 13]. Notably, *Callithrix* hybrids present a higher proportion of helminth infection rates than pure species [14]. In Rio de Janeiro, *Trypanosoma minasense* and *T. devei* were detected in *C. jacchus* at Tijuca National Park [15], with *T. minasense* also identified in wild *Callithrix* sp. in Botanical Garden in captive marmosets at the Wild Animal Screening Center of Rio de Janeiro (CETAS-RJ) [16, 17]. Furthermore, marmosets – including *C. jacchus*, *C. penicillata*, and *C. geoffroyi* – can host *T. cruzi* [8], the etiological agent of the zoonotic Chagas Disease. The detection of this parasite in *Sapajus nigritus*, at CETAS-RJ, highlights the necessity of epidemiological monitoring of marmosets, as they may become infected through shared facilities with other infected non-human primates (NHP) [17].

Trypanosomatids are protozoa distinguished by the presence of a single flagellum and a specialized organelle called kinetoplast [18]. Generally, the species within this group share a similar life cycle involving hematophagous insect vectors of the Reduviidae family, which transmit *T. cruzi*, *T. devei*, and *T. rangeli* [19–21]. Among these, *T. minasense* has a unique behavioral trait: a correlation between the circadian rhythm and parasitemia levels [22]. Another peculiarity is the size polymorphism observed in *T. cruzi* and *T. minasense* [16, 23, 24], which has been attributed to the wide range of hosts in both cases, and in the case of *T. cruzi*, the diversity of vectors [25]. The small size (17–22 μm), slender form, and a large, round subterminal kinetoplast are morphological traits widely accepted as diagnostic to *T. cruzi* [26]. In relation to *T. minasense*, the large form (26–48 μm) [16, 24] and an anteriorly positioned kinetoplast, a hallmark of the subgenus *Megatrypanum* [19].

Although several studies on hemoparasites have been conducted in the last century, the topic still lacks recent research due to logistical, ethical, and administrative issues involving blood and tissue collection from free-living NHP [27]. For instance, there is no data about some aspects of the biological life cycle of *T. minasense*, such as its vectors and true pathogenesis, although it is considered non-pathogenic in *C. jacchus* and *C. penicillata* [15]. Expanding research into all facets of host-parasite cycles is a core principle of the One Health concept [28, 29]. This approach treats wildlife, agricultural, and urban environments as a single interconnected ecosystem, recognizing that pathogens and diseases affect the whole [28, 29]. Continuous monitoring to gather data on pathogens is essential for developing prevention strategies for both species conservation and public health [28, 29]. Furthermore, marmosets serve as ideal model organisms for human disease studies due to their susceptibility to infectious agents and their geographical and phylogenetic proximity to humans [7, 30].

Our main objectives were to monitor trypanosomatid infection in free-living and captive marmosets (*Callithrix sp.*) in the Seropédica municipality, specifically at the Universidade Federal Rural do Rio de Janeiro (UFRRJ) campus, and the Wild Animal Screening Center of Rio de Janeiro (CETAS-RJ).

## Materials and Methods

### Study Area, Sampling Procedures, and Period

**Fig. 1:**
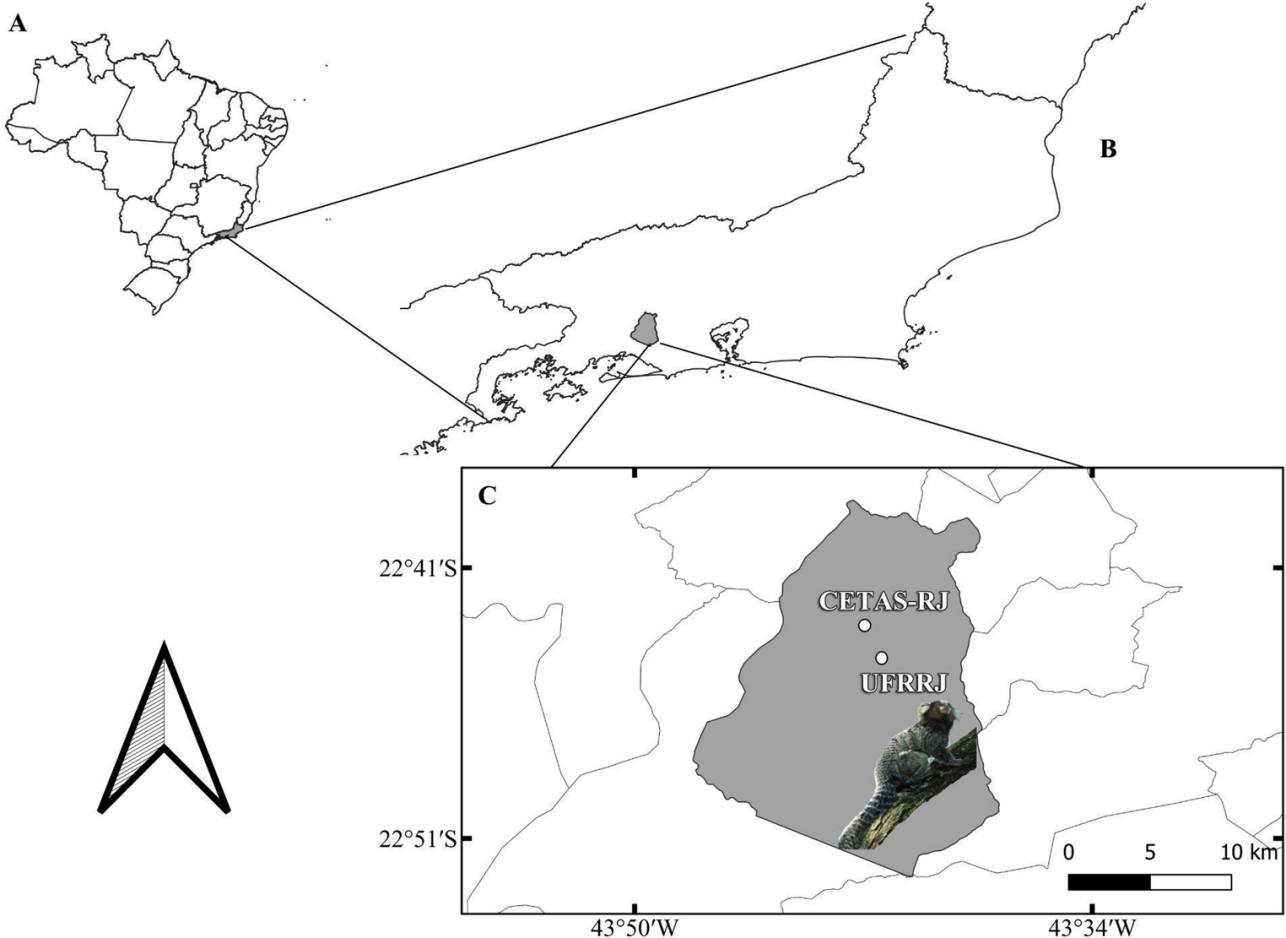
Marmosets sampling sites: A-Brazil map, B-Emphasis on the state of Rio de Janeiro, C-Seropédica municipality highlighting the two sampling locations: Wild Animal Screening Center (CETAS-RJ) and the Universidade Federal Rural do Rio de Janeiro campus (UFRRJ). Photo of a free-living *Callithrix* sp. at UFRRJ campus by Juliana de Moura Medeiros

The study was conducted in the city of Seropédica, one of the 13 municipalities of the Rio de Janeiro Metropolitan Area, which has 80,596 inhabitants (IBGE 2022). Additionally, there is an estimated daily circulating population of 10,000 individuals on the Universidade Federal Rural do Rio de Janeiro (UFRRJ) campus. Sampling was carried out in the Wild Animal Screening Center of Rio de Janeiro (CETAS-RJ) (22°45’38’’S, 43°42’28’’W) and the UFRRJ campus (22°45’47’’S, 43°41’18’’W). These sites are approximately 3 km apart (Fig. 1). The CETAS-RJ is a governmental organization responsible for the treatment and reintroduction of rescued wild animals. It is located in the Mário Xavier National Forest, a conservation unit, which holds 493 ha of native Atlantic Forest. The UFRRJ campus has 3,024 ha comprising natural or managed green areas, with small forest fragments and open grasslands.

Ten captures were carried out from January 2018 to November 2018, totaling 90 h of sampling effort. Three Tomahawk traps (18 × 18 × 60 cm) baited with bananas were set from 8:00 am to 5:00 pm to capture wild animals. These traps were placed on a bamboo platform hoisted in tree branches, four meters above the ground. Captive animals from CETAS-RJ were previously isolated in individual cages by the staff before biological sampling.

The procedures for animal capture and handling were approved by the Ethics Committee on Animal Use from Institute of Biological and Health Sciences at the Universidade Federal Rural do Rio de Janeiro (number 004/2018) and authorized by Sisbio/ICMBio/MMA (licenses 20435-2 and 59088-1).

### Biological Sampling

After capture, the traps were covered with a black cloth to reduce animal stress. Marmosets were weighed while inside the traps, and the weight of the trap and cloth was subsequently subtracted. Individuals were manually immobilized against bottom grid of the cage using leather gloves. Sedation was induced via intramuscular injection with Ketamine Hydrochloride (10 mg/kg) and muscle relaxant (Midazolam 0.05 mg/kg). Once sedation was confirmed, individuals were microchipped (Microchip partners^®^ dimensions 12 × 2.1 mm, ca. 0.006 g) in the dorsal interscapular region to identify recaptures [31].

Most sampled marmosets exhibited an intermediate range of coat pattern between *C. jacchus* and *C. penicillata*, suggesting that they were hybrids [5]. Consequently, they were identified as *Callithrix* sp. (*C*sp) followed by a subsequent numeral corresponding to the sampling order (Supplementary Table).

Approximately 1 ml of blood was collected from each individual by puncturing the inguinal arteriovenous plexus (24G needles; 0,55 × 20 mm). The blood was immediately transferred to 5 mL Vacutainer tubes with EDTA. Three blood smears were prepared per individual, fixed by immersion in absolute methanol for 10 min, and stained with 10% Giemsa solution in distilled water for 30 min [32].

Following data collection, the animals were released at their respective capture sites or returned to their designated cages (for captive marmosets) as soon as they showed signs of full reflex recovery.

### Optical Microscopy

Blood smears were analyzed using an Olympus BX45^®^ microscope, with 1000x magnification. Morphometric measurements of *Trypanosoma* were performed using D’Cell^®^ software, following the methodology of Hoare [19].

### DNA Extraction, Polymerase Chain Reaction, and DNA Sequencing

DNA was extracted from 150 µL of whole blood samples using the phenol-chloroform method [33]. The quantity and quality of the extracted DNA were estimated on a 0.8% agarose gel using Lambda DNA (Promega^®^) as a molecular weight marker and via NanoDrop 2000 (Thermo Fisher Scientific^®^).

For the amplification of a 700 to 800 base pairs (bp) region of the *Trypanosoma 18 S rRNA* ribosomal gene, the primer set 18ST nF2 (5’-CAA CGA TGA CAC CCA TGA ATT GGG GA-3’) and 18ST nR3 (5’-TGC GCG ACC AAT AAT TGC AAT AC-3’) was used [34]. Polymerase Chain Reactions (PCR) were prepared to a final volume of 25 µL, following the standardized protocols [16]. A positive control (*Trypanosoma vivax* GenBank^®^ MH184514), and a negative control (nuclease-free water instead of DNA) were included to evaluate potential contamination. PCR was carried out in a Thermocycler (MyGene^®^ Series Peltier Thermal Cycler Model Mg96+) with the thermal cycling profile described by reference [16]. PCR products were analyzed by electrophoresis on a 2% agarose gel stained with ethidium bromide (10 mg/mL) at 100 V for 90 min, including a 100 bp DNA Ladder. Products were purified using CleanSweep™ PCR Purification Product kit (Thermo Scientific^®^) and sequenced via the Sanger method at ACTGene Análises Moleculares Ltda. A minimum of 10 ng/µL in each amplicon for sequencing was required by the ACTGene protocol. The sequences have been deposited in GenBank^®^ (https://submit.ncbi.nlm.nih.gov/subs/genbank/).

### Phylogenetic Analysis

To assess the phylogenetic relationships between the generated sequences and those deposited in GenBank^®^, we used the Mega11^®^ software [35] and the BLAST^®^ tool. Phylogenetic analysis was conducted with the Maximum Likelihood method using the General Time Reversible (GTR) model [36], and branch support was evaluated with 1,000 bootstrap replications. In addition to the sequences generated in this study, we included sequences from *T. minasense* (AB36411.1, AB36412.1, MH578594.2, MH578593.1) and other *Trypanosoma* species (*T. grayi* KF546526.1, *T. theileri* KF924257. 1, *T. thomasbancrofti* KT728396.1, *T. avium* KT728401.1, *T. rangeli* AJ012414.1, *T. cruzi* AF228685.1 and X53917.1, *T. ralphi* TCC2218, *T. brucei* M12676.1, *T. vivax* MH184515.1, *T. sapaensis* AB242822.1, *T. talpae* AJ620545.1, *T. microti* AJ009158.1, *T. musculi* AJ223568.1, *T. rabinowitschae* AY491765.1, *T. wauwau* TCC1878, *T. boissoni* U39580.1, *T. triglae* U39584.1, *T. evansi* AJ009154.1, *T. simiae* AJ009162.1). *Leishmania infantum* (KF302746.1) and *Crithidia fasciculata* (Y00055.1) were included as outgroup.

### Statistical Analysis

To evaluate the statistical difference between groups and diagnostic methods, Fisher’s exact test [37, 38] and a binomial logistic regression model [39, 40] were employed. Odds ratio (OR) and 95% confidence interval (CI) were calculated [40]. The significance level was set at *P* < 0.05. All analyses were performed with RStudio version 2026.01.1 + 403.

## Results

A total of 25 marmosets were sampled: 12 free-living marmosets captured on UFRRJ campus (*C*sp49, *C*sp50, *C*sp51, *C*sp52, *C*sp56, *C*sp57, *C*sp63, *C*sp64, *C*sp65, *C*sp66, *C*sp67, and *C*sp68), and 13 captive marmosets from CETAS-RJ (*C*sp43, *C*sp44, *C*sp45, *C*sp46, *C*sp47, *C*sp48, *C*sp53, *C*sp54, *C*sp55, *C*sp58, *C*sp59, *C*sp61, and *C*sp62).

### Microscopy and Morphometric Analysis

**Fig. 2:**
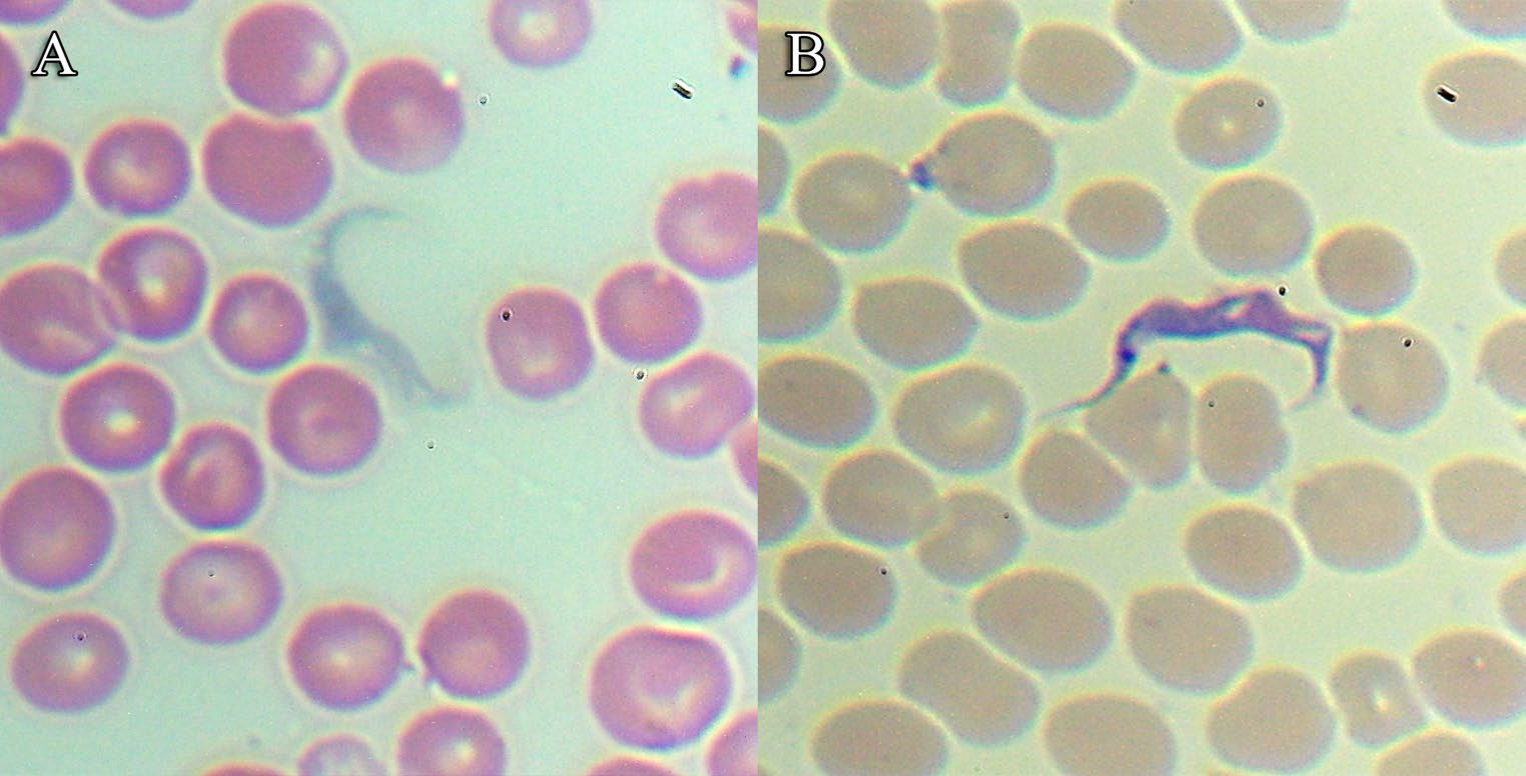
*Trypanosoma sp.* detected with light microscopy at 1000x magnification, in Giemsa-stained blood smear of two free-living marmosets captured on the UFRRJ campus in Seropédica, Rio de Janeiro Metropolitan area: A-*Csp*49; B-*Csp*64

The analysis of blood smears by optical microscopy detected *Trypanosoma* in two of the twenty-five marmosets sampled, both from the UFRRJ: *C*sp49 and *C*sp64 (Fig. 2). Only a single parasite was observed across the three blood smears prepared for each of these individuals.

The morphometric measurements (Table 1) were within the range described for *T. minasense*. Specifically the key variables to the species—TL values of 33.11 and 41.74 μm (species range 24.14–52.11 μm) and PK distances of 8.44 and 9.42 μm (species range 6.8–15 μm) were consistent with previous reports [16, 24].

**Table 1:** Morphometric data of *Trypanosom*a sp. identified by microscopy in blood smears from two *Callithrix* sp. individuals sampled on the UFRRJ campus, Seropédica, Metropolitan area of Rio de Janeiro. All measurements are expressed in micrometer (µm)

ID	*N*	PK	KN	PN	NA	FF	TL	NL	B
*Csp*49	1	8,44	9,31	17,75	7,17	8,28	33,11	3,13	1,86
*Csp*64	1	9,42	11,4	20,82	7,31	13,36	41,74	3,13	1,86

Sample identification (ID), number of parasites (N), distance between the posterior end and the kinetoplast (PK), distance between the kinetoplast and the nucleus (KN), distance between the posterior end to the center of the nucleus (PN), distance from the center of the nucleus to the anterior extremity (NA), free flagellum length (FF), total length (TL), nucleus length (NL), and body width at the nucleus level (B)

### Polymerase Chain Reaction for *Trypanosoma* Diagnosis

PCR amplification was positive in ten samples, including the two that were positive by microscopy (UFRRJ *Csp*49 and *Csp*64). Additionally, eight samples that were negative by microscopy tested positive by PCR: three from CETAS-RJ (*C*sp45, *C*sp53, and *C*sp62) and five from UFRRJ (*C*sp50, *C*sp51, *C*sp52, *C*sp57, and *C*sp67). Amplicons exhibited a molecular weight of approximately 700 bp on a 2% agarose gel, consistent with the positive control.

### DNA Sequencing and Phylogenetic Analysis

**Fig. 3:**
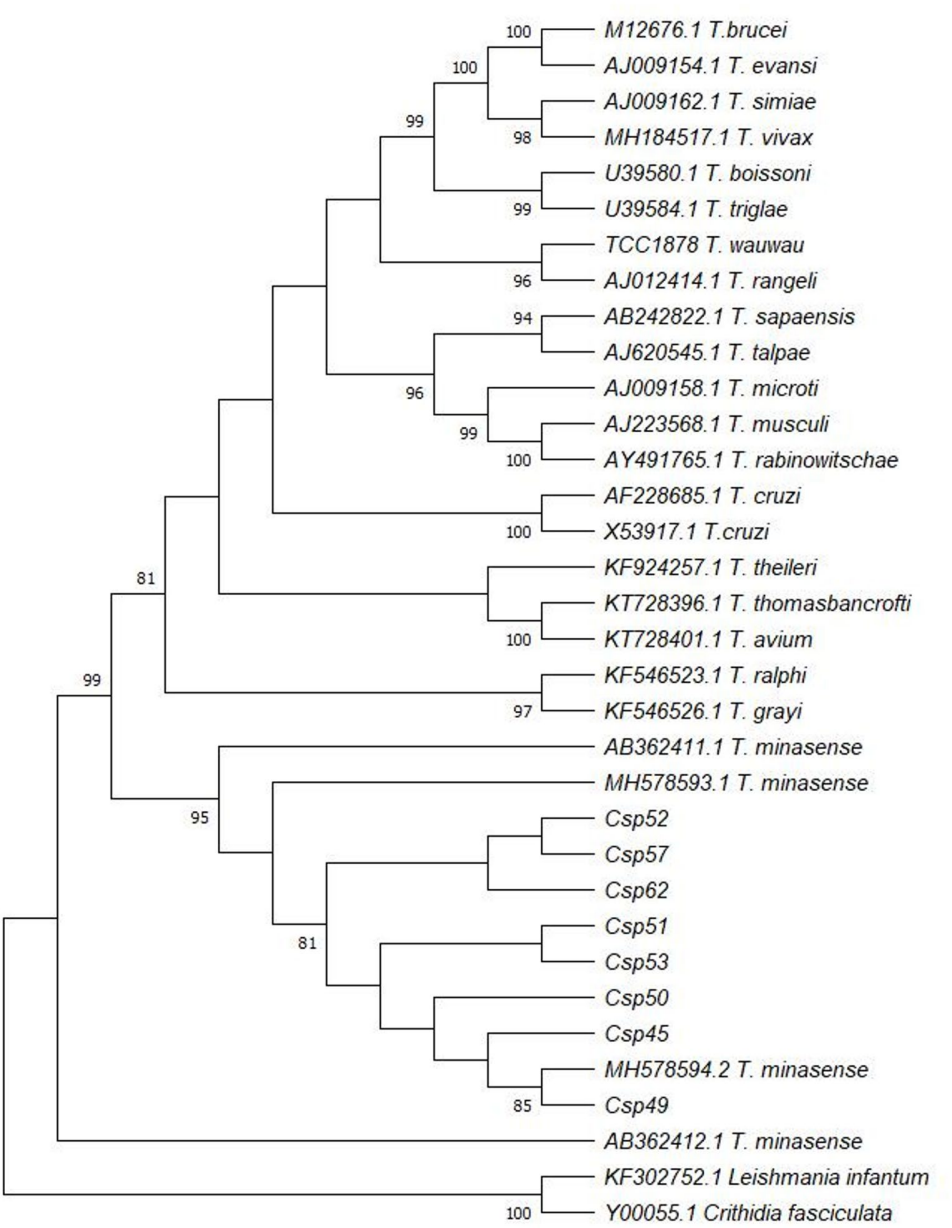
Maximum Likelihood phylogenetic tree based on the alignment of *18 S rRNA* gene sequences from *Trypanosoma* sp. identified in eight *Callithrix* sp. samples from CETAS-RJ and UFRRJ campus, Seropédica, Rio de Janeiro. DNA sequences generated in this study are designated as *Csp* and compared with *Trypanosoma* sequences from GeneBank^®^. *Leishmania infantum* and *Crithidia fasciculata* were used as outgroup. Bootstrap values are expressed as percentages based on 1000 replications using the general time reversible (GTR) model

The alignment of the *Trypanosoma* sp. *18 S rRNA* amplicons via BLAST revealed high identity with the sequence AB362411.1 deposited in GenBank^®^ [41], corresponding to *T. minasense* (subgenus *Megatrypanum*). An identity of 98% for *Csp*45, *C*sp49, and *Csp*50, while the remaining five positive samples showed 99%. The generated sequences were deposited in GenBank^®^ under accession numbers MW234441.1 (*Csp*45), MW234442.1-MW234446.1 (*Csp*49-*Csp*53), MW234447.1 (*Csp*57), and MW234448.1 (*Csp*62). Amplicons from *Csp*64 and *Csp*67 were not sequenced due to low DNA concentration. In the phylogenetic analysis, all eight sequences clustered with *T. minasense*, forming a distinct clade supported by a 95% bootstrap value (Fig. 3).

### Statistical Analysis

The probability of *Trypanosoma* infection was higher in free-living marmosets (58.3%) than in captive individuals (23.1%). Although the odds ratio indicated that free-living marmosets were 4.67 times more likely to be infected with *T. minasense* compared to captive individuals (95% CI: 0.83–26.2), the difference was not statistically significant following Fisher’s exact test (*P* = 0.082), and by logistic regression (*P* = 0.08). The detection probability using PCR was significantly higher (40%) than in microscopy (8%), with the odds ratio indicating that molecular genetics was 7.67 times more likely to achieve success compared to microscopy (95% CI: 1.48–39.7). The difference was significant in both tests, Fisher’s exact test (*P* = 0.012) and logistic regression (*P* = 0.015).

## Discussion

Ten out of 25 marmosets sampled in Seropédica, within the Rio de Janeiro Metropolitan area, tested positive for *Trypanosoma minasense*. The low detection rate by microscopy may be attributed to low parasitemia, consistent with observations for *T. minasense* infections [22, 24, 42]. Furthermore, the timing of blood collection may have influenced the negative microscopy results, since *T. minasense* parasitemia follows a circadian rhythm, peaking after 4:00 pm [22]. In this study, all samples were collected between 9:00 am and 2:00 pm. Low or undetected parasitemia is associated with reduced infective competence — the parasite’s ability to infect hosts and vectors—which can be influenced by the host’s species, age, nutritional status, and overall health [43]. Consequently, the low parasitemia observed may explain the low infection rates, particularly among captive marmosets, which tested negative for *Trypanosoma* sp. in the blood smear analysis.

Molecular genetic methods, such as PCR and DNA sequencing, are considered the most effective methods for the detection and identification of parasites [27, 42]. The of molecular genetics was confirmed in this study, which yielded ten positive results for *T. minasense* (40%) compared to only two detections via microscopy (8%). This difference was statistically significant (Fisher’s exact test *P* = 0.012; logistic regression *P* = 0.015), demonstrating that molecular genetics was 7.67 times more successful in identifying parasites. While the concurrent use of optical microscopy and molecular genetics may provide more robust results [16, 44, 45] our findings suggest that molecular genetics should be prioritized for the monitoring of *Trypanosoma* sp.

Free-living marmosets presented a higher *T. minasense* infection rate (58%) compared to captive individuals (23%), considering both methods. Although free-living marmosets exhibited a 4.67-fold higher probability of infection, this difference was not statistically significant (Fisher’s exact test *P* = 0.082; logistic regression *P* = 0.08). However, the p-value is close to the significance threshold (*P* < 0.05), suggesting a potential trend toward group differentiation, and perhaps the statistical results have been influenced by the limited sample size. The infection rate observed in free-living marmosets from UFRRJ was higher than that previously reported for the Rio de Janeiro Botanical Garden (33%, using both methods) and Tijuca National Park (47%, via microscopy) [15, 16]. Similarly, the infection rate for captive animals in our study exceeded the 12% previously recorded at CETAS-RJ using molecular genetics analysis [17]. According to different studies in the Rio de Janeiro Metropolitan area, captive animals from CETAS-RJ consistently present lower infection rates than their free-living counterparts. Nevertheless, it is crucial to note the disparities in sample size across studies (Botanical Garden, *n* = 15; Tijuca National Park, *n* = 34; previous CETAS-RJ research, *n* = 8), may have influenced these comparative outcomes.

Although the vector of *T. minasense* remains unidentified [19, 41, 46], it is presumed that its transmission cycle involves a hematophagous insect, as is typical for trypanosomatids. Consequently, the environment at CETAS-RJ may influence this cycle, potentially through the absence or exclusion of suitable vectors. The marmosets held at CETAS-RJ were originally from various regions of Rio de Janeiro and other Brazilian states. Despite the lack of specific data regarding the origin of these seized animals or the precise duration they shared the same enclosure, these factors likely played a pivotal role in the observed infection rates. Furthermore, captive animals undergo routine veterinary medical health monitoring, a practice that may hinder parasite persistence. Conversely, high-density primate population in captivity are generally expected to facilitate pathogen prevalence, particularly such as those transmitted by mosquitoes [47]. However, the absence of *T. cruzi* in marmosets at CETAS-RJ, despite its detection in capuchin monkeys (*Sapajus nigritus*) [17], suggests the presence of significant transmission barriers. Given that *Callithrix* is a known host for *T. cruzi*, and proximity to infected animals in high-density small areas typically promotes transmission, the specific environment and medical veterinary controls at CETAS-RJ may be mitigating the parasite spread.

The dilution hypothesis, which proposes that species diversity reduce parasite prevalence and abundance within a community [48], may further explain this findings. According to this model, non-host or incompetent host species can inhibit the parasite circulation by regulating vector population through predation [48] or by acting as alternative blood-meal sources, therefore, reducing the encounter rate between vectors and competent hosts [49]. The abundance of birds within the relatively small CETAS-RJ facility could fit in these two scenarios. Furthermore, vector specialization on “focal” or optimal hosts can decrease infection rates in secondary host species [48, 49]. In this case, *S. nigritus* is considered the optimal hosts for *T. minasense* [50]. Given that they are present at CETAS-RJ and has shown higher *T. minasense* infection rates than marmosets [17], their presence may have diverted encounters away from the *Callithrix* population. Since there are no free-living capuchin monkeys in Seropédica, their captive presence likely altered the local transmission. Nevertheless, these considerations should be interpreted with caution, and further studies are required to evaluate the dilution effect in this present context.

Beyond the local dynamics of CETAS-RJ, our findings align with the One Health framework by highlighting the interconnectedness of the environmental integrity and the wildlife health [29, 48, 51]. Environment degradation can change the diversity and density of hosts, pathogens, and vectors [51]. Therefore, parasites prevalence serves as sentinel for environmental conservation [52]. While *T. minasense* is non-zoonotic and generally non-pathogenic [19, 46], and in this case, its transmission is linked to forest fragmentation [50]. Primate population often present infection by different *Trypanosoma* species, sometimes, with mixed infections [53, 54]. Thus, the presence of the non-zoonotic *T. minasense* may serve as sentinels, indicating a suitable environment for the pathogenic Chagas disease trypanosomatid (54,55). Traditionally, trypanosomatid screening is driven by public health concerns regarding Chagas disease (*T. cruzi*). However, focusing exclusively on zoonotic threats can create a unidirectional bias in One Health research [55]. Our data demonstrate that despite the increasing proximity of *Callithrix* sp. and human populations in Seropédica, currently, trypanosomatids do not pose an immediate threat to public health or local wildlife. Nonetheless, the presence of this species confirms that the environment supports trypanosomatid cycles, emphasizing the need for continuous monitoring to detect potential spillovers or shifts in pathogen dynamics.

Beyond clinical and veterinary perspectives, parasitism is an ecological event, defined by the intricate interplay between host, parasite, and environment [56]. Parasites are, in a broad view, organisms whose ecological niches are interconnected [56–58]. Therefore, infection does not inherently result in disease [56], and this is probably the case of the non-pathogenic *T. minasense* —a distinction particularly relevant for the non-pathogenic *T. minasense* [19, 46], one of the most basal species of the subgenus *Megatrypanum* [19]. Despite its long history, since being described by Carlos Chagas in 1908 [59], significant gaps remain regarding its life cycle. Future investigations focusing on vector identification, reservoir dynamics and transmission modes, supported by larger sample sizes, are essential to fully elucidate the factor driving the divergent infection rates observed between free-living and captive marmosets.

## Conclusion

The study identified *Trypanosoma minasense* in 40% of the marmoset, sampled in Seropédica, Rio de Janeiro, using morphometric, molecular, and phylogenetic analysis of the *18 S rRNA* gene. PCR detection proved significantly more effective than microscopy. Although free-living marmosets exhibited higher infection rates than captive individuals, likely due to the influence of captive management practices on transmission dynamics and forest fragmentation, the difference was not statistically significant. The presence of *T. minasense* serves as a biological indicator, confirming that the sampled environments maintain favorable conditions for trypanosomatid circulation.

## Supplementary Information

Below is the link to the electronic supplementary material.

Supplementary Material 1

## Data Availability

No datasets were generated or analysed during the current study.
