# Supplementary material for: Trypanosoma minasense Parasitism in Free-Living and Captive Invasive Marmosets (Callitrichidae, Primates) in Seropédica, Rio de Janeiro, Brazil
Source: Acta Parasitol. 2026 Jun 1;71(3):132. doi: 10.1007/s11686-026-01306-0 (PMC13226333; doi:10.1007/s11686-026-01306-0)
Supplement: Supplementary file 1 — Supplementary Material 1 [file 11686_2026_1306_MOESM1_ESM.docx]

**Supplementary table**

| **ID** | **Age** | **Sex** | **Weight (g)** | **Microscopy** | **PCR** | **GenBank**  **Code Sequence** |
| --- | --- | --- | --- | --- | --- | --- |
| **UFRRJ** | | | | | | |
| *C*sp49 | J | M | 240 | + | + | MW234442.1 |
| *C*sp50 | A | M | 320 | - | + | MW234443.1 |
| *C*sp51 | A | M | 320 | - | + | MW234444.1 |
| *C*sp52 | A | F | 360 | - | + | MW234445.1 |
| *C*sp56 | A | M | 420 | - | - | - |
| *C*sp57 | A | F | 430 | - | + | MW234447.1 |
| *C*sp63 | J | F | 200 | - | - | - |
| *C*sp64 | A | M | 360 | + | + | no |
| *C*sp65 | J | M | 190 | - | - | - |
| *C*sp66 | J | M | 200 | - | - | - |
| *C*sp67 | A | F | 320 | - | + | no |
| *C*sp68 | A | M | 250 | - | - | - |
|  |  |  | **Cetas-RJ** |  |  |  |
| *C*sp43 | A | F | 310 | - | - | - |
| *C*sp44 | A | M | 330 | - | - | - |
| *C*sp45 | A | F | 281 | - | + | MW234441.1 |
| *C*sp46 | J | F | 220 | - | - | - |
| *C*sp47 | J | M | 195 | - | - | - |
| *C*sp48 | A | F | 330 | - | - | - |
| *C*sp53 | J | M | 346 | - | + | MW234446.1 |
| *C*sp54 | J | M | 203 | - | - | - |
| *C*sp55 | J | M | 166 | - | - | - |
| *C*sp58 | A | F | 285 | - | - | - |
| *C*sp59 | A | F | 330 | - | - | - |
| *C*sp61 | A | F | 330 | - | - | - |
| *C*sp62 | A | M | 270 | - | + | MW234448.1 |

*Callithrix* sp. (*C*sp) hybrids among *C. jacchus* and *C. penicillata,* juvenile (J), adult (A), male (M), female (F), grams (g), positive for the parasite (+), negative for the parasite (-), sequence not obtained (no).
